# Preliminary Evaluation of Filtration Efficiency and Differential Pressure ASTM F3502 Testing Methods of Non-Medical Masks Using a Face Filtration Mount

**DOI:** 10.3390/ijerph18084124

**Published:** 2021-04-13

**Authors:** Charles Freeman, Reuben Burch, Lesley Strawderman, Catherine Black, David Saucier, Jaime Rickert, John Wilson, Sarah Ashley Bealor, Madison Ratledge, Sydney Fava, Brian Smith, Charlie Waggoner, Courtney Taylor, Abigail Nichols, Gregory Skaggs, Thomas Callans

**Affiliations:** 1 School of Human Sciences, Mississippi State University, Starkville, MS 39762, USA; cmb1356@msstate.edu (C.B.); sab589@msstate.edu (S.A.B.); mr1488@msstate.edu (M.R.); sef231@msstate.edu (S.F.); agn78@msstate.edu (A.N.); 2 Industrial and Systems Engineering, Mississippi State University, Starkville, MS 39762, USA; burch@ise.msstate.edu (R.B.); strawderman@ise.msstate.edu (L.S.); dns105@msstate.edu (D.S.); smith@ise.msstate.edu (B.S.); 3 Institute Clean Energy and Technology, Mississippi State University, Starkville, MS 39762, USA; rickert@icet.msstate.edu (J.R.); wilson@icet.msstate.edu (J.W.); waggoner@icet.msstate.edu (C.W.); 4 Workforce and Economic Development, Golden Triangle Campus, East Mississippi Community College, Mayhew, MS 39753, USA; ctaylor@eastms.edu; 5 Tampa Bay Buccaneers, Tampa, FL 33607, USA; GSkaggs@Buccaneers.nfl.com; 6 Department of Athletics, Mississippi State University, Starkville, MS 39762, USA; tcallans@athletics.msstate.edu

**Keywords:** filtration efficiency, fabric masks, COVID-19, textiles

## Abstract

Research surrounding the mandated use of non-medical fabric masks is inconsistent and often confusing when compared to the standard N95. A recently published standard from ASTM International and the Centers for Disease Control and Prevention attempts to normalize evaluation procedures. The purpose of this study is to conduct a preliminary evaluation of the new methods for testing filtration efficiency of masks outlined by ASTM International F3502, where results can be directly compared to standards outlined for non-medical fabric masks. Eleven consumer non-medical fabric masks were tested for filtration efficiency and airflow resistance using a face filtration mount in accordance with the newly released ASTM International standard for facial barriers. The mean FE% (SD) ranged from 0.46% (0.44) to 11.80% (2.76) with the 3-layer athletic mesh having the highest performance and the highest deviations. All the masks tested following the procedure failed to meet to minimum FE of 20%; however all masks performed below the minimum upper limits for airflow resistance. Using a non-medical fabric masks as the sole mitigation strategy may not be as effective, as previously reported. With efforts to standardize and regulate the non-medical fabric mask market, this study demonstrates a variety of currently available consumer mask products do not meet the minimum standards nor are these remotely close to the standards of surgical or N95 masks.

## 1. Introduction

According to the CDC [1] and WHO [2], masks work to mitigate and slow the spread of SARS COVID-19, yet direct scientific evidence is still lacking as to the effectiveness of filtration by masks. To date, the literature remains contradictory on the level of surgical and non-medical mask effectiveness [3,4,5]. Studies on surgical masks considered an improvement from the non-medical masks are inconclusive when using standardized methods of filtration efficiency testing methods [2,6,7,8,9]. For example, Kähler and Hain [6] indicate almost all the droplets from breathing and coughing pass through a surgical mask unhindered, providing little to no protection for the wearer or those within a four meter proximity. Engineering and fluid mechanics research methods provided a discipline-specific standardized procedure, yet results indicate only large droplet sizes filtered during breathing and coughing. In non-medical fabric mask studies, results are similarly disparate. Viola et al. [9] evaluated aerosol spread when using face shields, and masks indicate the droplets spread when breathing is more extensive than previously expected. Drewnick et al. [2] modified the procedure outlined in 42 CFR Part 84 Standard Procedures [10] for testing and certifying air-purifying and particulate respirator reporting filtration efficiencies between 10–90%, with high variability between the fabric-based non-medical masks. Mask fit filtration results are also highly variable across studies. Clapp et al. [11] reported significant variability across fitted filtration efficiencies of 25.5% to 80.9% across commercially available masks fitted to a wearer. As indicated by the prior discrepancies in the literature, further studies utilizing filtration standardized testing methods remain critical to understanding non-medical masks’ effectiveness better. To regulate what has quickly become the “wild west” of non-medical masks, the CDC, in partnership with ASTM International (formerly American Society of Testing Materials), recently released ASTM F3502 Standard Specification for Barrier Face Coverings [12]. The standard establishes testing methods and minimum performance requirements (20% filtration efficiency and <0.59 w.c.) for non-medical fabric masks. Therefore, the purpose of this study is to conduct a preliminary evaluation of the new methods for testing filtration efficiency of masks outlined by ASTM International F3502 [12], where results are comparable to standards outlined for non-medical fabric masks. Based on the current 42 CFR Part 84 Standard Procedures [10] for testing and certifying air-purifying and particulate respirators, masks’ testing using a face mount is currently unavailable. The current guidelines are for flat media (fabrics, filters, or flat medical masks) or a structured three-dimensional mask (N95) sealed to the plate for the TSI 8130A filtration testing instrument.

## 2. Materials and Methods

### 2.1. Testing Procedures Face Filtration Mount

**Figure 1:**
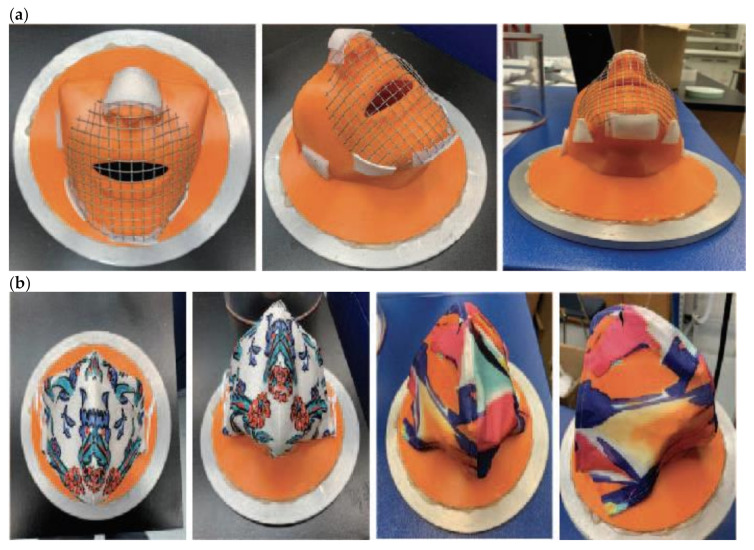
(**a**) 3D mask model with wire and double-sided tape adaptation to secure mask into place during testing. (**b**) 3D mask with mask samples showing mask placement during testing.

ASTM International F3502 [12] recommends the use of a sealed testing filtration holder and structured support to accommodate the flexible non-structured fabric masks is recommended. Based on these recommendations. Researchers designed and printed a 3-D face filtration mount (patent pending) adapted from the current CDC/NIOSH head and facial molds sized medium following average human head and mouth dimensions (Figure 1). CDC/NIOSH head form models are symmetric and represent the facial size and shape distribution of current U.S. respirator users (See CDC/NIOSH 2003 Anthropometric Survey Data: https://www.cdc.gov/niosh/npptl/topics/respirators/headforms.com for complete results, accessed on 9 April 2021). The nose and mouth on the face filtration mount match the average size and position for the chosen head form model (medium). Researchers fitted and secured samples to face filtration mount using double-sided tape at specific mask points of contact: bridge of the nose, under the chin, and along the jawbone. ASTM International F3502 [12] recommends a complete seal around the edges. However, this does not mimic the everyday mask use and wearer; therefore, this study sealed only at points of contact.

During initial face filtration mount testing, the facemasks tested on the mount were *suctioning* against the mouth with the airflow challenging the small area of fabric at the mouth. Results yielded high variability in filtration efficiency (FE) (±25% SD) and differential pressure (dP) (±0.75 SD) and further supported the decision not to completely seal the edges. After reset, a piece of metal screen supported the cloth masks above the mouth to prevent them from challenging only the fabric on the mouth. With the modification, readings’ consistency and reliability increased and better represented a mask’s performance on a face.

### 2.2. Testing Procedures Filtration Efficiency

Facemask evaluation used the TSI 8130A with a 3D printed face filtration mount intended to replicate a human face’s shape. These samples were tested for FE and dP in the TSI Model 8130A Automated Filter Tester with a 10 cm diameter pressure plate. The media samples during this test were held in place in the standard test chuck on the 8130A. The standard test chuck (11.5 cm tall with a diameter of 20.3 cm) has a flow area of 98.996 cm^2^, and pressure is managed through an electronic transducer with a range of 0–25 mm H_2_O. Samples rested 24 h in a conditioned laboratory at the temperature of 20 ± 2 °C (68 ± 4 °F) with relative humidity (RH) 65 ± 2%. Testing procedures used 42 CFR Part 84 Standard Procedures [10] to set the flow rates and conditions for testing and certifying air-purifying and particulate respirators. Aerosol production followed specifications and standards outlined with a Model 1,081,414 Oil Aerosol Generator and a Model 8118A Salt Aerosol Generator, representing different particle sizes. The oil aerosol specifications used a poly alpha olefin (PAO) oil aerosol with a mass median particle diameter of 0.33 µm and a count median particle diameter of 0.2 µm, required to validate FE specific to N95 masks. Secondly, sodium aerosol (NaCl) specifications included particles with a mass median particle diameter of 0.26 µm and a count median particle diameter of 0.075 µm, representing the average size of a SARS COVID-19 viral particle. Flow rates for both particle sizes followed standardized rates at ~85.0 L/min, much higher than the average breathing rate of 40.0–60.0 L/min. An increased flow rate not only conforms to standard methods outlined in ASTM International F3502 [12] but is representative of higher breathing rates during active periods [13]. Before testing with either aerosol, researchers recorded baseline readings for the face filtration mount without a facemask. Results from baseline testing indicate minimal particle obstruction or distortion in the testing chamber for either the PAO (0.32% FE, 0.0 dP) or the NaCl (0.08% FE, 0.0 dP). Testing procedures used *n* = 5 of each mask type to determine FE (%) and dP (w.c.). Due to the data’s non-normality, researchers conducted Kruskal-Wallis tests to determine if there were differences in outcomes across fabric types. For all tests, an alpha value of 0.05 was used. Post hoc analysis was conducted through a series of pairwise comparisons, with Bonferroni adjustment for multiple comparisons.

### 2.3. Products Tested

Per CDC [14] guidelines and recommendations by previous research [7,15], all masks have two or more fabric layers, except for the neck gaiter (mask 7), a one-ply knit. For testing purposes, the neck gaiter was double-layered to adhere to updated guidance requiring two-layer neck gaiters. Researchers selected eight knit masks and three woven masks. Three of the eight knit masks (masks 3, 4, and 11) selected had built-in filters, using fusible interfacing, melt-blown non-woven, and polyurethane open-cell foam, respectively. Three commercially available masks sold through name brand apparel retailers represent masks bought by the general public for occupational and everyday use (masks 8, 9, and 10). Lastly, two knit athletic performance masks represent those marketed and used for professional/collegiate and general public athletics (masks 2 and 11). In addition to the primarily cotton masks, three selected masks contain a blend of natural and synthetic fiber (cotton, viscose, polyester, nylon, and spandex) in the outer and lining fabrics. Five of the masks selected contained all synthetic fibers (polyester, nylon, and spandex) for outer and lining fabrics, with two of those marketed explicitly for athletic performance. Researchers identified fiber content based on required manufacturers labeling, validated through lab testing following fiber identification methods from the American Association of Textile Colorists and Chemists (AATCC) and ASTM International.

Fabric structure and thread count/gauge assessments followed ASTM D3775-17e1 Standard Test Method for End (Warp) and Pick (Filling) Count of Woven Fabrics [16] and ASTM D8007-15(2019) Standard Test Method for Wale and Course Count of Weft Knitted Fabrics [17]. Following ASTM 3776 Standard Test Methods for Mass Per Unit Area (Weight) of Fabric [18], researchers calculated fabric weights in grams per square meter (GM^2^) using a 100 cm^2^ sample cutter. GM^2^ was calculated for all mask fabrics, including outer, inter, and lining. GM^2^ for multi-layer masks were combined for a total GM^2^ rating for each of the masks to be used for statistical analysis. Researchers followed ASTM D1777—96 Standard Test Method for Thickness of Textile Materials [19] to determine mask (all layers) thickness. Using an automatic thickness gauge with a digital compression apparatus, researchers averaged five random measurements for each of the five samples/masks and used total mask thickness for statistical analysis. N95 and surgical masks are excluded from this study because they are omitted for testing in the new standard. See Table 1 for a complete list of masks with fiber and fabric characteristics.

## 3. Results

Of all the masks tested, only one reported an FE above 10% for either aerosol particles (PAO and NaCl). For the one mask reporting above 10%, the standard deviation was also the highest at 2.76, indicating high variability. Despite a limited sample (*n* = 5), researchers do not anticipate higher FE with more samples based on the reasonably consistent SD across mask types. Contrary to prior studies, FE results across mask types remained consistent and within the 10% FE range and well below the 95% standard for N95 or 70% for surgical masks. Data indicates the testing methods suggested are well suited to establish consistency in FE measurements; however, the standards outlined for non-medical fabric masks may be unrealistic. Overall, there exist significant differences between mask types and FE and dP for both the PAO and NaCl. Based on these results, post hoc analyses are discussed for those masks indicating significant differences, and non-significant masks are excluded from the discussion. Table 2 contains a complete listing of means and standard deviations for FE and dP, for both PAO and NaCl.

### 3.1. Filtration Efficiency and Differential Pressure

Filtration efficiency was significantly different based on mask type when the NaCl aerosol was used (χ2(10, *n* = 55) = 46.248, *p* < 0.001). Post hoc analysis showed that the filtration efficiency of mask 9 was significantly lower than masks 1, 3, 8, and 11. Additionally, the filtration efficiency of fabric 10 was significantly lower than masks 3, 8, and 11. Results were similar with the PAO aerosol, with mask type having a significant effect on filtration efficiency (χ2(10, *n* = 55) = 39.934, *p* < 0.001). Post hoc analysis showed that the filtration efficiency of masks 9 and 10 was significantly lower than masks 6 and 8.

Differential pressure was significantly different based on mask type (χ2(10, *n* = 55) = 44.228, *p* < 0.001). Post hoc analysis showed that mask 10 has a significantly lower differential pressure than masks 1, 3, and 7. Additionally, masks 4, 9, and 11 had significantly lower differential pressures compared to mask 1. When the PAO aerosol was used, the differential pressure was again significantly different based on mask type (χ2(10, *n* = 55) = 30.274, *p* < 0.001). The differential pressure of mask 4 was significantly lower than masks 6, 7, and 8.

### 3.2. Effect of Material Weight and Thickness

There are multiple significant correlations between measures, as shown in Table 3. For both aerosol types, filtration efficiency increased as all other measures increased. Filtration efficiency correlated positively with both fabric weight and thickness. Therefore, it is essential to evaluate the fabric characteristics when selecting non-medical fabric masks. There are no significant reported differences between the fabrication types (knit, woven, non-woven) and FE, and therefore was not explored further. Of note, masks with higher thread/loop counts per 10 cm^2^ have the highest reported weights and thicknesses.

## 4. Discussion

While there is much debate on mask-wearing effectiveness, research methods allowing for direct comparisons remain limited. Therefore, it is crucial to understand that all masks are not equal, and despite recent research and product marketing, are not providing protection levels comparable to N95 respirators or surgical masks. However, due to the variety of materials and construction methods, discerning the properties, efficiency, and difference in one mask’s protection levels over the other is increasingly difficult. As peer-reviewed mask research has evolved since early March 2020, more robust studies and methods are being published to validate early-stage mask research. This study was created to evaluate non-medical fabric masks following the recently released ASTM International F3502. The current testing standard and recommendations are all provided by agencies based in the United States. There is current guidance from the WHO that is in line with the CDC recommendations on public health guidance.

Similarly, ASTM International standards for filtration are similar to ISO. However, ISO has not yet released a standard method for testing filtration efficiencies of non-medical fabric masks, whereas ASTM International recently released the standards used in this study. For this study’s scope, researchers applied the guidance and testing methods currently available, and as agencies issue new guidance on the international level, further research in support of these regulations will be needed.

According to 42CFR84 [10], N95 masks must meet a minimum 95% FE when challenged with 0.3 µm with a flow rate of 85.0 L/min and have a differential pressure no higher than 1.15 w.c. for exhalation. ASTM International [15] standards for surgical masks should exceed 95% FE and a dP of no more than 0.90 under similar testing conditions. For non-medical fabric masks, ASTM International [12] set standards at 20% FE and no more than 0.59 w.c. airflow resistance (dP). Results from this study indicate samples fell well below the NIOSH [10], Association Française de Normalisation (AFNOR) [20], and ASTM International [12,15] thresholds for FE and dP at standardized flow rates, which indicates higher breathability even during maximum exertion. However, none of the masks tested under standardized conditions came close to the 20% FE threshold. The highest FE reported at 0.3 µm, and 85.0 L/min is 6.52% FE (mask 8) and a dP of 0.07 w.c., with the lowest-performing mask (mask 9) at 0.46% FE and 0.04 w.c. dP. This study’s results are notably lower than prior research reporting FE for masks of 5–80% [3,5,7,21,22,23]. While results from this study report much lower FE, dP remains similar to prior research for similar mask types indicating airflow resistance methods are adequate, despite the inconsistency of FE reporting. While FE rates for the masks differed from prior research, it should be noted none of the studies mentioned above utilized and closely followed the testing conditions and procedures outlined for recently released certification of non-medical fabric masks.

Results from this study support prior research by Viola et al. [9] and Kähler and Hain [6], reporting most if not all of the respiratory particles are passed through loosely fitted surgical and non-medical fabric masks. While these studies examined airflow using thermal and fluid dynamics methods, this study supports their findings of significant respiratory particle passthrough when wearing loosely fitted surgical and non-medical fabric masks. Their findings are further supported by the lower dP scores for masks from this study, indicating sufficient airflow resistance. On the surface, results from this study could infer the ineffectiveness of masks; however, masks provide a critical disruptor to directional airflow when viewed in conjunction with the prior studies. In addition to filtering out a small percentage of particles, using a mask breaks up laminar airflow and, with impaction, creates turbulent airflow, thereby limiting the distance particles can travel away from an infected individual. Results from this study provide support to most current research involving airflow and filtration of surgical and non-medical masks.

For health care, occupational activities, especially those with higher levels of exertion and breathing, indicate that masks wearing masks can provide a level of protection, albeit not at a point where physical distancing is not followed. While there was a significant difference between some of the masks for FE and dP, the overall results for all of the masks do not support making a recommendation for a particular type of non-medical fabric mask. Results support reviewing the minimum standards set for masks and shifting the focus and conversation to the proper fitting and securing of non-medical fabric masks for maximum airflow disruption. However, wearing a mask, even at high exertion levels, does not affect breathability and, even with low FE, plays a crucial role in disputing the laminar airflow. Please see Table 4 for a summary of key findings and application to current and future research.

## 5. Conclusions

Throughout the SARS COVID-19 pandemic, research from across disciplines has worked to provide understanding and results to academia and the general public at an unprecedented pace. Due to this pace, the public and decision-makers witnessed the scientific method and testing, reporting, and validation cycle. This exposure has the indirect side effect of distrust with the science and constantly shifting guidelines and recommendations, especially for people’s activities. Of critical importance for health care providers is non-medical fabric masks worn by patients are not providing adequate filtration efficiency to prevent the spread of the virus, despite prior research with results above the 20% minimum. According to this study’s results, the combined filtration efficiency of two individuals wearing non-medical fabric masks remains well under 25%. This study indicates that many of the current commercial options are not sufficient to meet the new standards.

Based on these results and the CDC and WHO’s mitigation strategies, patients and health care providers wearing masks are only effective when used in conjunction with physical distancing and proper hygiene. Within the medical settings, physical distancing is very difficult with patient interactions; however, all parties’ wearing of masks may lead to a false sense of security and protection, especially by the non-medical staff with direct interactions with patients. Health care workers are being infected at an alarming rate, and with the introduction of multiple increased contagion variants, the risk will only continue to rise, despite vaccinations. Results from this study support wearing a mask to provide critical obstruction of laminar airflow and limits the distance away from an infected person a SARS COVID-19 particle travels. However, when using masks, it remains critical for people to understand the masks’ limitations and have the awareness that they do not solely provide a line of defense against the spread of or infection from the virus. Lastly, as the message from the federal health agencies is beginning to consolidate and become more consistent, health care providers with information from this study should increase mask messaging efforts to patients and peers as a comprehensive public health strategy.

## 6. Patents

The Face Filtration Mount for Automated Filter Tester has been disclosed to Mississippi State University (Disclosure #2020.1150) for patent filing.

## Figures and Tables

**Table 1:** Characteristics of Masks used in the Study.

Mask Layers	Available to Purchase?	Fabric Structure	Fiber Content	Thread/Loop Count (Per 10 cm^2^)	Mask Total Mass (g/m^2^)	Mask Total Thickness (mm)
Mask 1 Outer	N	Tricot	82% nylon 18% spandex	293	296	1.05
Mask 1 Lining	-	Single knit	87% cotton 13% polyester	153	-	-
Mask 2 Outer	Y	Double knit	93% polyester 7% spandex	135	431	1.50
Mask 2 lining	-	Raschel warp knit	92% polyester 8% spandex	217	-	-
Mask 3 Outer	Y	Double weft knit	100% polyester	195	278	0.97
Mask 3 lining	-	Single knit	50% viscose 50% cotton	170	-	-
Mask 4 Outer	Y	Raschel warp knit	100% polyester	170	281	1.21
Mask 4 inter-lining	-	Non-woven	100% polyester	n/a	-	-
Mask 4 lining	-	Raschel warp knit	100% polyester	209	-	-
Mask 5 Outer	Y	Plain Weave	100% cotton	310	277	0.62
Mask 5 lining	-	Single Knit	96% polyester 4% spandex	192	-	
Mask 6 Outer	N	Single Knit	89% cotton 11% spandex	229	322	1.25
Mask 6 lining	-	Single knit	87% cotton 13% polyester	153	-	
Mask 7 Outer	Y	Single Knit	83% polyester 17% spandex	195	192	0.46
Mask 8 Outer	Y	Double weft knit	100% polyester	212	320	0.99
Mask 8 lining	-	Double weft knit	100% polyester	211	-	-
Mask 9 Outer	Y	Plain Weave	100% cotton	245	241	0.61
Mask 9 lining	-	Plain Weave	100% cotton	276	-	-
Mask 10 Outer	Y	Plain Weave	100% cotton	324	206	0.40
Mask 10 lining	-	Plain Weave	100% cotton	324	-	-
Mask 11 Outer	Y	Double knit	100% polyester	144	439	2.01
Mask 11 inter-lining	-	open cell foam	100% polyurethane	n/a	-	-
Mask 11 lining	-	Double weft knit	77% nylon/23% spandex	234	-	-

N, no; Y, yes.

**Table 2:** Filtration Efficiency, Differential Pressure, and Q Factor Means and Standard Deviations.

Mask	FE Mean PAO %	FE Mean NaCl %	FE SD PAO %	FE SD NaCl %	dP Mean PAO w.c.	dP Mean NaCl w.c.	dP SD PAO w.c.	dP SD NaCl w.c.
Mask 1	3.15	8.48	0.97	0.77	0.06	0.12	0.01	0.01
Mask 2	2.74	6.48	0.86	1.89	0.04	0.08	<0.01	<0.01
Mask 3	3.95	10.0	1.26	1.11	0.05	0.09	<0.01	0.02
Mask 4	3.32	5.36	0.57	0.46	0.03	0.05	<0.01	0.01
Mask 5	2.48	5.64	0.45	1.17	0.05	0.07	<0.01	0.01
Mask 6	5.63	6.76	1.30	0.63	0.07	0.06	0.02	0.01
Mask 7	3.48	5.88	0.60	2.12	0.07	0.10	0.01	0.02
Mask 8	6.52	8.99	1.03	0.62	0.07	0.07	0.02	0.01
Mask 9	0.46	2.57	0.44	0.33	0.04	0.05	0.01	0.01
Mask 10	1.73	3.43	0.82	0.24	0.06	0.05	0.01	0.01
Mask 11	4.23	11.80	1.86	2.76	0.04	0.05	0.02	0.02

Note: For the scope of this study and based on the literature reviewed, a *natural log* (ln) Q factor. FE, filtration efficiency; PAO, poly alpha olefin; SD, standard deviation; dP, differential pressure; w.c., water column.

**Table 3:** Pearson Correlation Values between Measures when Using NaCl Aerosol.

	Differential Pressure NaCL (w.c.)	Differential Pressure PAO (w.c.)	Fabric Weight NaCl (g/m^2^)	Fabric Weight PAO (g/m^2^)	Fabric Thickness NaCl (mm)	Fabric Thickness PAO (mm)
Filtration Efficiency (%)	0.398 *	0.644 *	0.557 *	0.309 *	0.624 *	0.368 *
Differential Pressure (w.c.)	-		−0.047	−0.191	−0.076	−0.215
Fabric Weight (g/m^2^)	-		-		0.927 *	0.927 *

* *p*-Value < 0.5.

**Table 4:** Summary of Key Findings and Application to Research.

Key Findings	Applications to Current Research
Use of Standard Test Method	Methods of testing outside of standards for N95 and Surgical Masks limit the applicability of the results Following a standardized method provides an accurate assessment of effectiveness of fabric masks Procedure is repeatable and applicable to all types of non-medical fabric masks
Substandard Filtration Efficiency	Filtration efficiencies for non-medical fabric masks when tested under similar conditions as N95 and surgical are less than 12% effective General guidance from health organization is 20% minimum Differential pressure is below benchmarks indicating the masks are not hindering breathing
Guidance for Public Health Messaging	With lower filtration efficiencies than previous reported, masks alone will not mitigate the spread of COVID-19 Provide verified filtration efficiency information to the health sectors and general public to encourage physical distancing and mask wearing

## Data Availability

The data that support the findings of this study are available from the corresponding author, upon reasonable request.
